# The Role of Predictive Biomarkers in Endocervical Adenocarcinoma: Recommendations From the International Society of Gynecological Pathologists

**DOI:** 10.1097/PGP.0000000000000755

**Published:** 2021-02-09

**Authors:** Tjalling Bosse, Sigurd Lax, Nadeem Abu-Rustum, Xavier Matias-Guiu

**Affiliations:** Department of Pathology, Leiden University Medical Center, Leiden, The Netherlands (T.B.); Department of Pathology, General Hospital Graz II, Styrian Hospital Corporation and Medical University of Graz, and Johannes Kepler University, School of Medicine, Linz, Austria (S.L.); Department of Surgery, Gynecology Service, Memorial Sloan Kettering Cancer Center, New York, New York (N.A.-R.); Hospital Universitari Arnau de Vilanova and Hospital U de Bellvitge, Universities of Lleida and Barcelona, IRBLLEIDA, IDIBELL, CIBERONC, Lleida, Spain (X.M.-G.)

**Keywords:** Endocervix, Adenocarcinoma, Predictive factors, Recommendations

## Abstract

To review the scientific evidence related to predictive biomarkers in cervical adenocarcinoma (ADC). The authors reviewed the literature regarding predictive biomarkers in cervical ADC. There were several limitations: (1) there is an overlap between predictive and prognostic biomarkers, as the vast majority of patients are treated with anticancer strategies; (2) in many studies and clinical trials, cervical ADC patients are included in a large series of patients predominantly composed of cervical squamous cell carcinomas; and (3) in most of the studies, and clinical trials, there is no distinction between human papillomavirus (HPV)-associated and HPV-independent cervical ADCs, or between various histologic subtypes. Results obtained from a small group of studies confirm that cervical ADCs exhibit distinct molecular features as compared with squamous carcinomas, and that there are different molecular features between different types of cervical ADCs. Promising areas of interest include *ERBB2 (HER2)* mutations and PD-L1 expression as predictive biomarkers for anti-HER2 treatment and immunotherapy, respectively. To date, no definitive data can be obtained from the literature regarding predictive biomarkers for cervical ADC. Clinical trials specifically designed for endocervical ADC patients are required to elucidate the predictive value of *HER2* mutations and PD-L1 expression. The distinction between HPV-associated and HPV-independent cervical ADCs as well as early involvement of pathologists in the design of future clinical trials are needed to identify new predictive biomarkers in cervical ADC.

Cervical cancer ranks fourth in incidence and mortality in women 1. The incidence is particularly high in developing countries. Ninety-five percent of cases are caused by persistent human papillomavirus (HPV) infection 2. Most cervical cancers are squamous cell carcinomas (SCC) but cervical adenocarcinoma (ADC) has been increasing in both true and relative incidence 3,4 and in developing nations and institutions where cervical screening is well established may be seen in 25% of published series 5.

The updated WHO 2020 classification system recognizes the importance of distinguishing between HPV positive and negative tumors (designated as HPV associated and HPV independent, respectively), and subdivided the various histologic types under these 2 major groups of cervical ADC 6,7.

In this article, the authors reviewed the literature regarding prognostic and predictive factors in cervical ADC. Conventional prognostic factors (such as Silva patterns of invasion) are mentioned in other manuscripts. The authors identified several limitations:

1. There was an important overlap between predictive and prognostic biomarkers, as the vast majority of patients that were included in the studies had been treated with standard of care therapies, including chemoradiation therapy.
2. In many studies and clinical trials, cervical ADC patients were included in a large series of cases predominantly composed of SCC patients.
3. In most of the studies, and clinical trials, there was no distinction between HPV-associated and HPV-independent tumors. Moreover, the vast majority of the studies did not distinguish between histologic subtypes of cervical ADC.

A prognostic biomarker provides information about the patients’ overall cancer outcome, regardless of the therapy, whereas a predictive biomarker gives information about the response to a specific therapeutic intervention. In addition, a predictive biomarker (such as ER and HER2/neu in breast cancer) can be a potential target for therapy 8.

ISGYP established a multidisciplinary panel of members (one practicing clinician and 3 pathologists) that provide care to endocervical carcinoma patients to act as expert reviewers for the recommendations developed. A systematic literature review of relevant studies published between January 2014 and February 2020 was carried out using the MEDLINE database for combinations of the keywords related to the topic (endocervical cancer, adenocarcinoma, molecular, genetics, predictive, targeted therapy, immunotherapy, prognosis, HPV). The literature search was limited to publications in English. Priority was given to high-quality systematic reviews, meta-analyses, and randomized controlled trials, but studies of lower levels of evidence were also evaluated. The reference list of each identified article was reviewed for other potentially relevant articles. An initial document was written, and approved by the team members, and presented in power-point format during a satellite session in the USCAP meeting in Los Angeles in March 2020. With all suggestions taken into account, a manuscript was written and approved by all authors, and recommendations were submitted for the ISGYP membership for final approval.

## MOLECULAR FEATURES OF CERVICAL ADC

Several studies have addressed the integrated genomic and molecular characterization of cervical cancer including a small subset of ADC patients 8,9. A whole exome sequencing analysis of 115 cervical carcinomas with normal paired samples included 24 ADC cases and demonstrated *ELF3* and *CBFB* somatic mutations in 13% and 8% of cases, respectively 9. Moreover, the study confirmed *PIK3CA* (16%) and *KRAS* (8%) mutations, and showed that the PIK3CA/PTEN pathway was significantly mutated in the ADC group, which is relevant as this pathway is related to resistance for anti-HER2 therapies 10. TCGA performed an extensive molecular characterization, and included 32 ADC cases, some of them HPV negative 11. The study confirmed frequent *PIK3CA* and *KRAS* mutations, and *ERBB3 (HER3)* mutations. Frequent *BCAR4* amplification, putatively associated with anti-HER2 therapy was also detected, and frequent *CD274* amplification, putative targets for immunotherapy 11. A high-throughput genotyping platform, including 1250 known mutations in 139 cancer genes was used in 80 cervical tumors, including 40 SCC and 40 ADC cases 12, a vast majority of them associated with HPV. In this study, *PIK3CA* mutation rates did not differ significantly between ADC and SCC, whereas *KRAS* mutations were identified only in ADC 12. In a recent study in 154 cervical cancers, including 43 ADC, *KRAS* mutations were almost restricted to ADC patients, whereas *PIK3CA* mutations were more frequent in SCC. *TP53* mutations were more predominant in HPV-independent tumors, and *STK11* genomic alterations showed an association with lower overall survival 13.

A transcriptomic signature with molecular networks associated with SCC and ADC was characterized using oligomicroarray and pathway analysis 14. Some genes (*KRT17*, *IGFBP2*, *CALCA*, *VIPR1*) were differentially expressed in ADC and SCC. cDNA microarray analysis demonstrated differentially expressed genes specific for ADC (*CEACAM5*, *TACSTD1*, *S100P*, and *MSLN*) 15. In a different study 16, the authors assessed differential expression between ADC and SCC in a set of genes including those coding for 12-lipoxygenase (*12-LOX*), keratin 4, trypsinogen 2 (*TRY2*), Rh glycoprotein C (*RhGC*), collagen type V alpha 2, integrin alpha 5, integrin alpha 6, and *C-MYC*.

The clinicopathologic and prognostic relevance of *KRAS* mutation was assessed in a series of 876 invasive cervical carcinomas, which included 210 ADC cases 17. *KRAS* mutations were associated with HPV18, and more frequently detected in nonsquamous carcinoma, with a frequency of 7.3% in ADC. The presence of *KRAS* mutations was an independent predictor for tumor recurrence. Another study on cervical cancers, including 55 ADC, analyzed by mass spectrometry by assessing 171 somatic hot-spot mutations, identified *KRAS* mutations in 24% of ADC in comparison with 3% of SCC cases 18. In multivariate analysis, however, mutation status was not an independent predictor of survival.

Cervical ADC occasionally shows HER2 overexpression. In one study 19, 46% of ADC showed positive expression for EGFR and HER2, which significantly correlated with lymph node metastasis, stage, and short relapse-free survival. HER2 expression significantly correlated with tumor size. In a different study 20, HER2 expression was assessed in 13 cases of gastric-type ADC. Immunostaining was equivocal in six cases and *ERBB2 (HER2)* amplification was identified in one case. Relevance of *HER2* mutations will be discussed latter on.

A few studies have characterized HPV-independent ADC, including the gastric type 7. Banister et al. 21 analyzed a series of 212 SCC and 44 ADC, to characterize HPV-independent cervical cancers. HPV-associated tumors expressed E2F target genes and increased AKT/MTOR signaling while HPV-independent tumors had increased WNT/β-catenin and Sonic Hedgehog signaling. HPV-independent tumors showed a global decrease in DNA methylation, although there was some promoter-associated CpGs hypermethylation. HPV-independent tumors were enriched for nonsynonymous somatic mutations in *TP53, ARID*, as well as WNT, and PI3K pathways. Garg et al. 22 used next-generation sequencing for 161 unique cancer-driver genes for single-nucleotide and copy-number variations, gene fusions, and insertions/deletions in 14 cases. *TP53* was the most frequently mutated gene followed by *MSH6*, *CDKN2A/B*, *POLE*, *SLX4*, *ARID1A*, *STK11*, *BRCA2*, and *MSH2*. Abnormal p53 expression was observed in 9 cases by immunohistochemistry, whereas MDM2 gene amplification in 12q15 locus was seen in 2 cases that express normal p53 levels by immunohistochemistry. Hodgson et al. 23 performed a targeted massively parallel sequencing assay of 447 cancer genes and 191 regions across 60 genes for rearrangement detection in 56 ADC samples that included 45 HPV-associated and 11 gastric-type tumors. *KRAS*, *TP53*, and *PIK3CA* were the most commonly mutated genes, whereas alterations in *TP53*, *STK11*, *CDKN2A*, *ATM*, and *NTRK3* were significantly more common in gastric-type ADC. Tumors associated with adverse outcome, regardless of the histologic type, more commonly had alterations in *KRAS*, *GNAS*, and *CDKN2A*. The association between cervical ADC and *STK11* had been previously noted 24, based on the relationship between minimal deviation gastric-type ADC and Peutz-Jeghers syndrome.

As mentioned in previous publications, the pattern of ADC invasion, according Silva criteria has prognostic relevance. The Silva classification, however, is limited to HPV-associated cervical ADC. The molecular profile of cervical ADC has been associated with the Silva pattern of invasion, by using targeted sequencing with the Ion AmpliSeq Cancer Hotspot Panel v2 that assesses hotspot regions of 50 oncogenes and tumor suppressor genes 25. Mutations were frequently found in *PIK3CA* (30%), *KRAS* (30%), *MET* (15%), and *RB1* (10%). *PIK3CA, KRAS*, and *RB1* mutations were seen exclusively in pattern B or C subgroups, whereas *KRAS* mutations correlated with advanced stage at presentation.

Additional studies have shown molecular abnormalities in cervical ADC at different levels in genes such as *ZNF58S, SOX1, SOX17, EZH2*, and *L1CAM* 26–30.

## PREDICTIVE BIOMARKERS OF CHEMORADIOTHERAPY RESPONSE

A vast majority of patients with advanced cervical ADC are treated by combined radiation and chemotherapy. The mechanisms of resistance to these anticancer treatments are complex. There is a large amount of literature suggesting putative markers involved in response to treatment. It is not the intention of this section to provide a comprehensive review on this topic. The vast majority of the publications refers to cervical cancer in general, without emphasis on cervical ADC, which is important, as there are some studies suggesting poor response to radiation therapy in ADC, in comparison with SCC 31–33.

In one review of 19 publications on the mechanisms involved in resistance to radiation therapy 34, the authors identified a total of 23 biomarkers, which could be related to 6 biologic functions, such as apoptosis, cell adhesion, DNA repair, hypoxia, metabolism, pluripotency, and proliferation. In a different review of published studies 35, the authors identified 6 immunohistochemical markers with controversial correlation with chemoradiotherapy response (p53, p21, Ki67, EGFR, HER2, BCL-2), and 11 immunohistochemical biomarkers with positive correlation with chemoradiotherapy (HPV, pAKT, COX-2, nitric oxide synthase, HIF-1-alpha, HIF-2-alpha, VEGF, NF-kb, Ku80, EMMPRIN). Moreover, microarray studies have also suggested that the expression of sets of genes were associated with and without recurrence after radiation therapy 36–39.

Several processes and proteins have been related to cisplatin resistance in cervical cancer 40, including: (1) a reduction in the intracellular accumulation of platinum compounds (CTR1, multidrug resistance proteins, GSH), (2) increase in DNA damage repair, (3) inactivation of apoptosis (caspases, BCL family, NF-kb, p53 signaling), (4) activation of epithelial to mesenchymal transition, (5) and other mechanisms such as alteration in DNA methylation, microRNA profile, stemness, and stress response. D44v6, XRCC, and mTOR were also related to the prediction of sensitivity to platinum-type agents in neoadjuvant chemotherapy 40.

Some other biomarkers have been related to sensitivity to specific agents, such as CHFR in the prediction of sensitivity to paclitaxel, WRN in relation to sensitivity to CPT-11, and HIF-1α in the prediction of sensitivity to topotecan 40. Neoadjuvant treatment would provide a novel window of opportunity to study response and biomarker relationships. It would be helpful if pathologists develop a standardized approach to assess response to neoadjuvant treatments.

## TARGETED THERAPY

Different strategies have been proposed in the treatment of cervical cancer. Yet again, most studies and clinical trials do not consider ADC patients separately, so the information must be taken with caution.

Angiogenesis is a critical process in carcinogenesis and tumor progression. HPV oncoproteins play key roles in upregulating angiogenesis, through their effects on p53 degradation and inactivation of pRb, which lead to increased VEGF pathway and HIF-1-alpha expression 41. Angiogenesis has been successfully targeted in cervical cancer, as the results of the GOG 240 trials (including 310 patients with SCC and 86 with ADC) and subsequent trials were published 42–44. Since then, bevacizumab was approved by the FDA and became standard of care in a subset of patients with advanced cervical cancer. No predictive biomarker of antiangiogenic response has reached clinical practice.

Several other drugs and corresponding predictive biomarkers have been proposed 45,46. They include EGFR inhibitors 47–49 and PARP-1 inhibitors 50,51, because of the expression of EGFR 18 and presence of homologous recombination-related gene mutations 52 in cervical cancer. None of them, however, have reached clinical practice. Tisotumab vedotin, an antibody-drug conjugate targeting tissue factor has got encouraging results 53, but no specific predictive biomarker has been proposed.

A promising targeted therapy approach at present is *ERBB2 (HER2)* and *ERBB3 (HER3)*, the genes that encode for HER2 and HER3. As mentioned before, HER2 overexpression and *HER2* amplification were previously shown in cervical ADC. Somatic mutations in *ERBB2/3 (HER2/3)* were found in a wide range of cancers 54, and lead to constitutive HER2/3 activation. *HER2* mutations were detected in 4% to5.5% of cervical cancers 54,55. *PIK3CA* mutations represented one of the most frequent co-alterations in *HER2*-mutant cancers 56; and this is a problem, as PIK3CA mutations are known to result in resistance to anti-HER2 treatment 9. These preliminary studies have shown that a subset of patients with cervical cancer and HER2 inhibition achieved complete/partial response and stable disease in basket trials 54. In one study with 1015 patients with cervical cancer, *HER2* mutations were found in 4.5% ADC, but only in 2.1% SCC 56. *HER2* mutations frequently coexisted with *PIK3CA* or *KRAS* mutations. In that series of cases, 33 nonsynonymous somatic *HER2* mutations were detected, including 30 missense mutations and 3 in-frame deletions. Nineteen *HER2* mutations were located within the extracellular domain, four in the transmembrane domain, and 10 in the Kinase domain. The most prevalent mutation spot was S310F (6 cases), followed by A270S (5 cases). Among patients who were tested for both *HER2* gene mutations and overexpression/amplification, no concurrence of mutation and overexpression/amplification was found. A case report has shown successful result of HER2 inhibition in 1 patient with advanced cervical ADC with *HER2* amplification 57. It appears that HER2 inhibition can be an interesting tool for ADC patients with *HER2* mutation or amplification, and maybe with *BCAR4* amplification. A combined therapy targeting simultaneously *HER2* and *PIK3CA* has also been suggested 58. Pathologists have experience in the quality control of HER2 expression assessment 59,60. Interpretation of predictive biomarkers, such as HER2, has shown to be context specific, as seen in differences in criteria for breast and gastric carcinoma 61. Therefore, it is worth mentioning that there is still insufficient experience on how to score HER2 immunohistochemistry in the context of cervical ADC. Gynecologic pathology studies focusing on scoring and quantitating HER2 expression in cervical ADC should be encouraged.

## IMMUNOTHERAPY

The main objective of cancer immunotherapy is to enhance tumor antigen-specific immune responses that can target tumor cells. Many different studies have demonstrated that immunotherapy may be helpful in the treatment of a variety of tumors. The emergence of immune checkpoint inhibitors has opened a new door to cancer therapy.

Cervical cancer is a good candidate tumor for immunotherapy approaches. There are several reasons for this. Cervical cancer has a relatively high rate of tumor mutational burden 62, frequent amplification of immune targets 11, and frequent involvement of HPV. There is increasing evidence showing that immune checkpoint inhibitors may have a potential role in the treatment of virus-related cancers 63. It has been shown that HPV E7 may increase PD-L1 expression after transfection into cancer cells 64.

Immune checkpoints such as programmed death 1 (PD-1) and cytotoxic T lymphocyte antigen 4 (CTLA-4) are membrane-bound molecules, which are expressed on immune cells. Immune checkpoint inhibitors block the binding of immune checkpoint molecules to their ligands, reversing the inactivation of T cells, enhancing the immune response of T cells. These inhibitors may have a role in virus clearance and may have a greater effect in virus-associated cancers 63.

In a recent overview about the role of biomarkers for the prediction of response to checkpoint immunotherapy 65, it is shown that cervical cancer is frequently positive for PD-L1, and show a moderate mutational burden, with 5-6 mutations per megabase. Higher ratios of CD8+ tumor-infiltrating lymphocytes to CD4^+^ T regulatory cells have been associated with improved survival. Response rate of cervical cancer to checkpoint immunotherapy is within the range of 10% to 25%.

PD-L1 expression was assessed in 2 cohorts of primary cervical carcinomas (156 SCC and 49 ADC), and matched primary and metastatic tumors (96 SCC and 31 ADC) 66 using the E1L3N clone on an automated Ventana immunostainer. Tumor cells were designated positive when >5% of tumor cells were positive. Distinction was made between diffuse (throughout the tumor), or marginal (interphase between tumor and stroma). Scores were also calculated for PD-L1-positive tumor-infiltrating immune cells. SCC was more frequently positive for PD-L1 and contained more PD-L1-positive tumor-associated macrophages. Disease-specific survival was significantly worse in ADC patients with PD-L1-positive tumor-associated macrophages compared with ADCA patients without PD-L1-positive tumor-associated macrophages. No difference between primary and metastatic tumors was seen. In another study 67, PD-L1 (clone SP142), by combining intensity and percentage of positive cells, was expressed in 32 of 93 (34.4%) cervical carcinomas, including 2 of 12 (16.7%) ADC.

A meta-analysis including seven studies with 783 patients 68 also suggested that PD-L1 overexpression was associated with poor overall survival. The methodology was different, and the number of ADC cases was variable 69–71. One study including 127 samples was limited to ADC 72. The density of immune cells and expression levels were compared between the tumor cell groups and stroma, using digital image analysis. Expression of PD-L1 on tumor cells was found in 17.3% of the cases. A higher density of stroma-infiltrating lymphocytes and macrophages was found in PD-L1-positive tumors than in negative tumors. In this study, patients with PD-L1-positive tumors tended to experience longer survival. In one study with 97 patients, 7 of them ADC 73, PD-L1 expression correlated with tumor-infiltrating lymphocytes, and response to neoadjuvant chemotherapy.

Four phase 1 and 2 clinical trials assessed the value of check point inhibitors in cervical cancers. In 3 of them, ADC patients were included. In one of them 74, Ipilimumab was administered to 42 previously treated patients with cervical cancer, 13 of them with ADC. PD-L1 expression, as assessed by E1L3N clone, was negative in 20 patients, positive (10%) in 4, and positive (>10%) in additional 4 patients. There was partial response in 1 patient and stable disease in 10. PD-L1 expression was not predictive of therapeutic benefit and PD-L1 expression did not change during treatment. In the Keynote-028 trial 75, Pembrolizumab was administered to 22 previously treated patients with cervical cancer, including a single patient with ADC. PD-L1 expression, assessed by the 22C3 clone with a cutoff of >1% was positive in tumor cells in 18 cases, and in 6 cases in both tumor and stromal cells. There was partial response in 4 patients, and stable disease in 3 patients. Finally, in the Keynote-158 study 76, Pembrolizumab was administered to 98 patients with previously treated cervical cancer, including 5 patients with ADC. PD-L1 was assessed by the 22C3 clone, by using the combined positive score (CPS) (>1), which is a ratio of tumor lymphocytes and macrophages by the total of tumor cells. All ADC were positive (CPS > 1). The objective response rate was higher in patients with PD-L1-positive tumors. No responses were observed in patients with PD-L1-negative tumors, but the number of cases was too small to draw conclusions. After publication of the Keynote-158 trial, the Food and Drug Administration (FDA) approved pembrolizumab for patients with recurrent or metastatic cervical cancer with disease progression on or after chemotherapy, whose tumors express PD-L1 (CPS of 1 or higher), as determined by the FDA-approved companion test, by 22C3 clone. Until new data is provided (with additional clinical trials with other drugs, a significant proportion of ADC patients, and assessment the different antibodies available as best companion diagnostic test), it seems reasonable to give support to the current FDA-approved guidelines.

There are several ongoing phase III randomized trials (Keynote-826,-NCT03635567, BEATcc-NCT03556839, GOG3016-NCT03257267) with several immune checkpoint inhibitors in women with metastatic and/or recurrent cervical cancers.

Tumor microenvironment can have an impact on prognosis. Several studies have shown an improved survival associated with an increase in the number of tumor-infiltrating lymphocytes 77,78. There is an association between a high number of intratumor CD8^+^ lymphocytes and absence of lymph node metastasis 79.

However, the perspectives of immunotherapy in cervical carcinoma go beyond checkpoint inhibitors. TIM3 is a candidate target that is expressed on immune cells, and contributes to immune tolerance 80. TIM3 is expressed in cervical tumors, and may be associated with tumor progression 81. Other interesting strategies are therapeutic vaccines and adoptive cell therapies.

### Recommendation 1

Expert gynecologic pathologists should take the lead in developing robust guidelines for testing and scoring HER2 and PD-L1 immunohistochemistry to facilitate standardization in clinical trials. It is strongly recommended to interpret and report predictive biomarkers to response of treatment in endocervical ADC in correlation with well-established pathologic parameters.

### Recommendation 2

Until specific recommendations are validated for endocervical ADC, prediction of immunotherapy response criteria is identical to that for squamous cervical cancer. At present, PD-L1 immunohistochemistry (CPS of 1 or higher), as determined by the FDA-approved companion test, by 22C3 clone, is recommended for pembrolizumab treatment of patients with recurrent or metastatic cervical cancer with disease progression on or after chemotherapy.

### Recommendation 3

With the exception of PD-L1, and based on the lack of scientific evidence at the present time, no other biomarker is recommended for the prediction of treatment response in endocervical ADC.

## COMBINATION OF TREATMENTS

Radiation therapy is an effective treatment for local tumor control, but may also elicit a systemic effect, which can lead to an antitumor effect that can kill cancer cells outside of the radiation filed. This been reported as the Abscopal effect 82,83. The mechanisms responsible for the Abscopal effect are not well understood, and the immune system is thought to play an important role. It has been suggested that immune modulation from PD-1/PD-L1 inhibitors and radiation therapy through nonredundant pathways may contribute to synergistic activity, which is the basis of combination of radiation therapy and immunotherapy. Some studies show increased PD-L1 positivity in tissue samples, after radiation therapy 84.

## CLINICAL TRIALS

To date, no definitive data can be obtained from the literature regarding predictive biomarkers for treatment response in cervical ADC (Table 1). So far, clinical trials have predominantly included patients with SCC. Clinical trials specifically designed for endocervical ADC patients are encouraged to elucidate the predictive value of *HER2* amplification and mutations as well as PD-L1 expression. Involvement of pathologists in designing these clinical trials is needed to identify new predictive biomarkers in cervical ADC. Although clinical trials are not the main domain of gynecological pathologists, it is important to emphasize that their involvement is needed for ideal methodologic strategy. Pathologists should take the lead in developing robust guidelines for testing and scoring HER2 and PD-L1 immunohistochemistry to facilitate standardization in clinical trials. Given the relative rarity of ADC, an international multi-institutional effort is required to move this field forward, particularly to recruit enough patients with HPV-independent ADC to achieve the appropriate statistical power for an HPV-independent arm.

**TABLE 1:** Summary of main prognostic/predictive biomarkers in cervical adenocarcinoma

Biomarker	Predictive/prognostic	Target	Evidence
*BCAR4* amplification	Predictive	*HER2*	Low
*KRAS* mutation	Prognostic	*KRAS*	Strong
*HER2* amplification	Predictive/prognostic	*HER2*	Moderate
*HER2* mutation	Predictive	*HER2*	Strong
PDL-1 expression	Predictive/prognostic	Immune checkpoint inhibition	Moderate

### Recommendation 4

Clinical trials specifically designed for HPV-associated and HPV-independent endocervical ADC patients are strongly encouraged to elucidate the predictive value of some biomarkers (ERBB2 PD-L1, and others). Trials combining the unbalanced number of patients with ADC (including HPV-independent disease) and SCC may yield results not necessarily applicable to endocervical adenocarcinoma patients.

### Recommendation 5

Involvement of expert gynecologic pathologists in the design of future clinical trials is strongly recommended to appropriately identify new predictive biomarkers in cervical adenocarcinoma.
